# Transitioning *Public Health Nutrition* to Open Access

**DOI:** 10.1017/S1368980022000088

**Published:** 2022-02

As readers of *Public Health Nutrition*, you will know the journal is transitioning to Open Access from January 2022 onwards and as 2022 commences it seems a good time to review the change and what it means for us.

For the *Public Health Nutrition* community, Open Access means articles are freely and permanently available to all, facilitating further research and adoption of research findings. For authors, Gold Open Access articles published by Cambridge University Press & Assessment received three times the use in the first 12 months relative to subscription articles, and more citations across 2 years for both humanities and social sciences; and science, technology and medical fields.

While some Open Access journals have been of questionable quality and appear to be a means for authors prepared to pay for publication to publish whatever they want, PHN has not changed the rigorous peer review and publication process that we have always had, maintaining the high quality of articles.

The requirement for researchers to make their research findings freely available will also increase with the adoption of Plan S which has the following objective: ‘With effect from 2021, all scholarly publications on the results from research funded by public or private grants provided by national, regional and international research councils and funding bodies, must be published in Open Access Journals, on Open Access Platforms, or made immediately available through Open Access Repositories without embargo.’ PHN signalled its commitment to Open Access by becoming a Transformative Journal in October 2020, and now, in January 2022, makes the transition to being fully OA.

The imposition of article processing charges is not intended to limit access to publication by authors with limited budgets. As detailed on the PHN website, authors whose institution has signed a Read and Publish agreement with Cambridge University Press & Assessment receive either a full or partial waiver of the charges.

https://www.cambridge.org/core/journals/public-health-nutrition/public-health-nutrition-open-access-frequently-asked-questions

When the corresponding author is based in a Research4 Life Group A country, article processing charges will be waived, and a 50 % waiver will be granted where the corresponding author is based in a Research4Life Group B country. Individual cases will also be assessed, and in rare cases when authors and their institutes can clearly demonstrate their inability to pay, charges will be waived. In this way, we maximise the opportunity for all authors to publish in PHN, regardless of their funding availability.

And what has happened to journal submissions because of the transition to Open Access? The figure below shows the cumulative number of manuscripts submitted each month over the last 6 years. The green line for 2021 clearly shows a drop in the rate of submissions after March when PHN changed to Open Access, but this has been smaller than anticipated, and is a typical trend for all OA flips. In fact, since the ScholarOne site transitioned to OA ahead of the 2022 volume, PHN has received submissions from seventy-one counties, with the number of submissions being substantially greater than what was forecasted, which is very encouraging. These are exciting times for the journal, but our experience with the transition so far affirms our ambition that PHN will maintain its place as the destination for dissemination of research and scholarship aimed at understanding nutrition-related public health achievements, situations and problems around the world.

**Figure:**
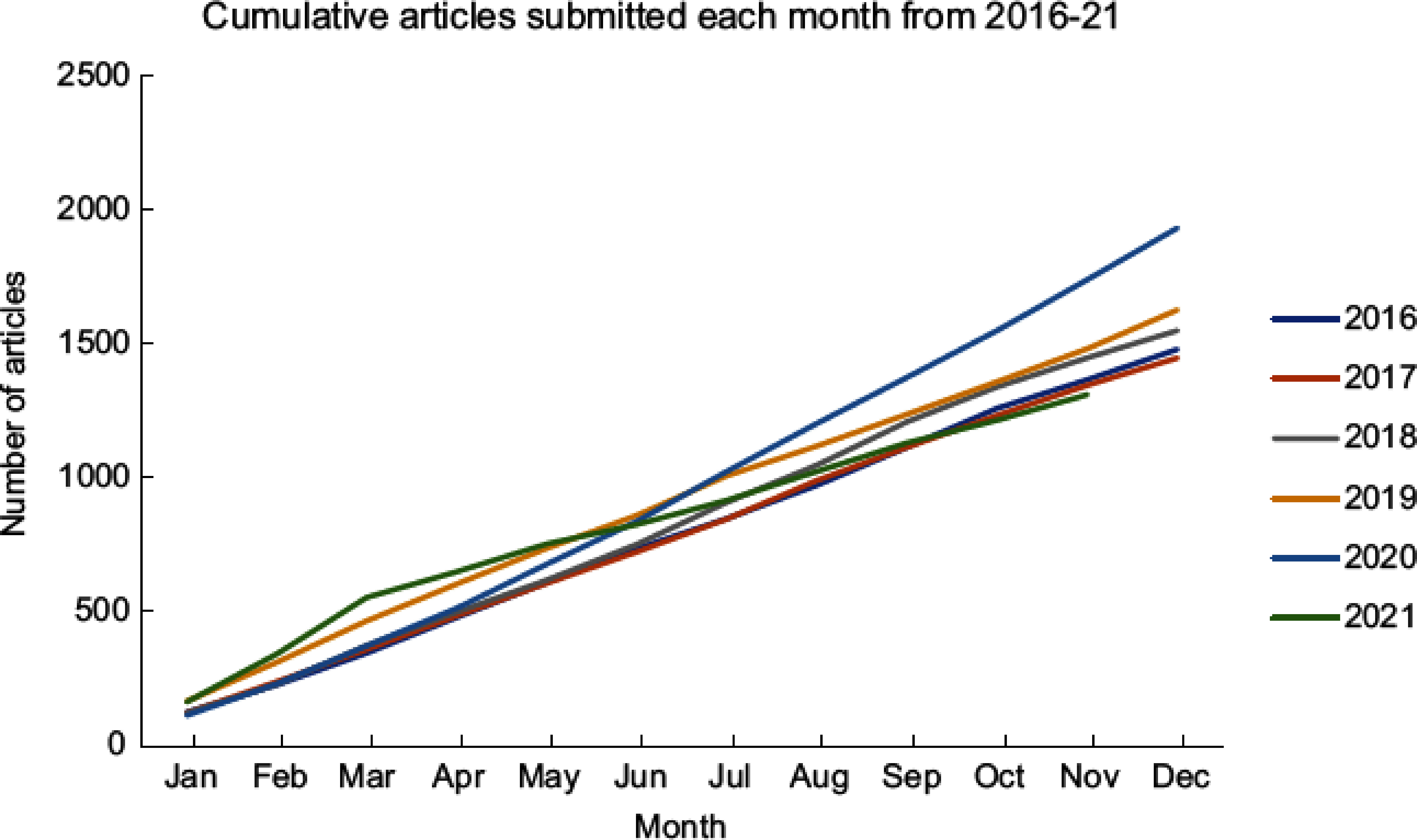

I would like to thank the team at Cambridge University Press & Assessment, all the reviewers, authors and readers for continuing to make PHN the success it is today and wish you all the best for 2022.

